# Supplementary material for: Volatile Metabolome and Aroma Differences of Six Cultivars of Prunus mume Blossoms
Source: Plants (Basel). 2023 Jan 9;12(2):308. doi: 10.3390/plants12020308 (PMC9863704; doi:10.3390/plants12020308)
Supplement: Supplementary file 1 [file plants-12-00308-s001.zip › plants-2120765-supplementary.pdf]

Figure S1. TIC of volatile compounds in six cultivars of P. mume blossoms by GC-MS

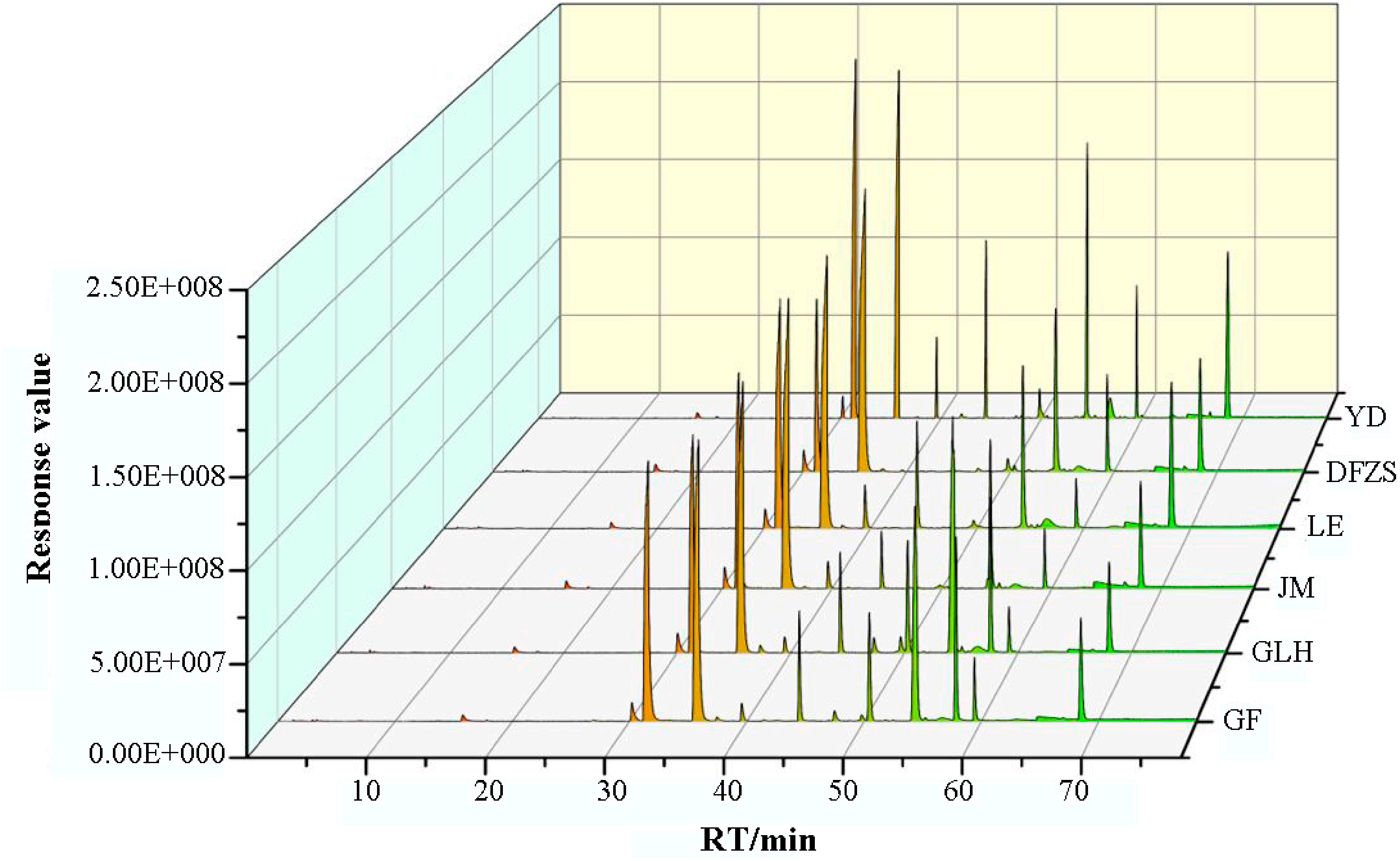

Table S1. Contents of 89 detected volatile compounds in six cultivars of *P. mume* blossoms

Number	Compound Name	CAS-number	Content μg/g					
			YD	JM	GLH	LE	DFZS	GF
1	Dimethyl sulfide	75-18-3	0.04±0.01	-	0.04±0.03	0.06±0.08	0.02±0.01	0.03±0.01
2	Acetic acid, methyl ester	79-20-9	0.15±0.03	0.26±0.04	0.27±0.05	0.09±0.01	0.21±0.03	0.15±0.02
3	Hexanal	66-25-1	0.21±0.02	0.16±0.05	0.08±0.03	0.11±0.02	0.05±0.02	0.14±0.03
4	o-Xylene	108-38-3	0.04±0.04	-	-	-	-	-
5	unknown 1		0.04±0.04	0.08±0.01	0.03±0.03	0.05±0.03	0.03±0.03	0.05±0.02
6	2-Hexenal, (E)-	6728-26-3	0.59±0.07	0.82±0.22	0.34±0.06	0.23±0.03	0.44±0.21	0.52±0.18
7	unknown 2		0.02±0.03	0.07±0.02	0.05±0.02	0.07±0.01	0.04±0.01	0.06±0.03
8	Styrene	100-42-5	0.01±0.04	-	0.09±0.03	0.00±0.01	0.02±0.01	0.07±0.04
9	1-Hexanol	111-27-3	0.09±0.02	0.05±0.01	0.03±0.01	0.05±0.03	0.05±0.02	0.06±0.01
10	3-Hexen-1-ol*	544-12-7	0.14±0.04	0.05±0.01	0.05±0.04	0.08±0.03	0.06±0.02	0.08±0.03
11	2-Hexen-1-ol（E)	928-95-0	0.15±0.13	0.21±0.04	0.11±0.04	0.07±0.03	0.11±0.05	0.14±0.04
12	Nonanal	124-19-6	0.26±0.04	0.23±0.03	0.36±0.04	0.19±0.03	0.25±0.04	0.54±0.09
13	Acetic acid	64-19-7	0.07±0.06	0.09±0.02	0.09±0.03	0.06±0.02	0.04±0.01	0.10±0.05
14	6-Hepten-1-ol, 2-methyl-*	67133-86-2	0.08±0.05	0.10±0.04	0.01±0.02	-	0.05±0.03	0.04±0.03
15	Benzaldehyde	100-52-7	157.00±9.21	112.00±4.21	108.02±6.00	126.77±9.83	62.61±7.59	149.91±8.05
16	2-Nonenal*	2463-53-8	0.07±0.06	-	0.10±0.05	-	-	0.03±0.04
17	Fenchol*	1632-73-1	0.12±0.09	-	0.05±0.10	-	-	-
18	Bornyl acetate*	76-49-3	0.07±0.06	-	0.00±0.01	-	-	0.01±0.02
19	Methyl benzoate	93-58-3	172.36±7.23	207.26±6.85	166.90±5.90	210.76±11.67	212.20±6.35	157.84±8.81
20	(E)-α-Elemene*	5951-67-7	0.11±0.09	-	0.09±0.04	-	-	-
21	1-Nonanol	143-08-8	0.08±0.02	0.18±0.03	-	0.07±0.01	0.17±0.04	0.11±0.04
22	Ethyl benzoate	93-89-0	0.27±0.11	1.13±0.38	2.38±0.49	0.54±0.27	0.47±0.14	1.21±0.79
23	4-Allylanisole	140-67-0	0.50±0.12	0.13±0.05	0.22±0.08	0.11±0.10	1.20±0.15	0.19±0.07

24	Benzyl formate	104-57-4	-	0.05±0.04	0.08±0.07	0.09±0.07	-	0.07±0.09
25	Borneol*	507-70-0	0.11±0.02	0.17±0.10	0.09±0.02	0.07±0.03	0.12±0.03	0.13±0.03
26	Benzyl acetate	140-11-4	15.99±1.21	8.69±2.15	4.34±0.38	11.29±1.62	0.84±0.15	5.80±1.16
27	Carvone*	99-49-0	0.09±0.02	-	-	-	-	-
28	unknown 3		-	-	0.01±0.03	0.05±0.03	0.09±0.03	-
29	(Z,E)-α-Farnesene*	26560-14-5	0.17±0.05	-	-	-	-	-
30	Methyl salicylate	119-36-8	0.12±0.01	0.50±0.06	0.30±0.08	0.36±0.11	0.25±0.03	0.41±0.07
31	α-Farnesene*	502-61-4	2.67±0.82	-	-	-	-	-
32	unknown 4		-	0.24±0.10	0.41±0.26	0.21±0.05	0.04±0.04	0.19±0.06
33	3,4-Dimethoxytoluene*	494-99-5	0.28±0.03	0.08±0.04	0.13±0.05	0.51±0.07	0.13±0.05	0.19±0.12
34	2,4-Decadienal*	2363-88-4	0.05±0.04	-	-	-	-	-
35	Carveol*	1197-07-5	0.07±0.09	-	-	-	-	-
36	unknown 5		0.30±0.06	0.21±0.04	0.14±0.05	0.10±0.06	0.19±0.03	0.11±0.05
37	Benzyl alcohol	100-51-6	41.41±5.73	13.81±2.66	19.68±3.19	23.67±3.49	6.82±1.33	22.65±2.73
38	Butyl benzoate	136-60-7	0.23±0.04	0.38±0.16	0.25±0.05	0.40±0.04	0.57±0.09	0.25±0.06
39	(Z)-Cinnamaldehyde	57194-69-1	-	-	0.17±0.03	-	0.07±0.12	0.14±0.02
40	Phenylethyl Alcohol	1960/12/8	0.08±0.03	0.12±0.01	0.13±0.07	0.14±0.02	0.12±0.01	0.12±0.02
41	unknown 6		0.06±0.04	0.05±0.02	0.06±0.02	0.03±0.01	0.04±0.03	0.02±0.01
42	unknown 7		0.06±0.08	0.07±0.02	0.02±0.03	0.06±0.03	0.06±0.02	0.03±0.01
43	Isoamyl benzoate	94-46-2	0.23±0.03	0.97±0.19	0.31±0.11	0.56±0.11	1.60±0.25	0.42±0.13
44	Creosol	93-51-6	0.29±0.02	-	0.13±0.18	-	-	-
45	Phenylpropyl acetate	122-72-5	0.46±0.08	0.34±0.07	3.85±0.63	0.27±0.06	0.14±0.03	3.21±0.31
46	trans-β-Ionone	79-77-6	0.45±0.12	0.41±0.03	0.24±0.16	0.22±0.06	0.15±0.08	0.26±0.08
47	Dihydro-β-ionol	3293-47-8	0.22±0.05	0.09±0.02	0.03±0.06	0.07±0.03	0.01±0.03	0.07±0.05
48	unknown 8		0.09±0.05	0.13±0.02	0.05±0.05	0.05±0.03	0.23±0.06	0.09±0.05
49	Pentyl benzoate	2049-96-9	-	0.30±0.04	0.24±0.11	0.24±0.05	0.46±0.07	0.17±0.07

50	Methyleugenol	93-15-2	5.11±0.57	1.53±0.38	4.83±0.30	1.97±0.67	2.60±0.58	2.06±0.33
51	Alkane1		4.03±0.53	1.97±0.48	-	1.46±0.65	1.19±0.50	-
52	3-Phenyl-1-propanol	122-97-4	0.18±0.11	-	1.42±0.35	-	0.07±0.22	1.21±0.21
53	(E)-Cinnamaldehyde	14371-10-9	0.91±0.29	0.26±0.09	27.52±7.41	0.29±0.16	1.89±0.31	25.09±2.48
54	unknown 9		0.12±0.02	0.22±0.05	-	0.07±0.03	0.17±0.14	0.00±0.01
55	unknown 10		0.11±0.03	0.02±0.03	0.06±0.06	0.07±0.03	0.12±0.07	0.06±0.03
56	(E)-Methyl cinnamate	1754-62-7	0.24±0.03	0.41±0.08	0.40±0.08	0.30±0.05	0.85±0.10	0.28±0.06
57	unknown 11		0.09±0.01	-	-	0.02±0.05	-	-
58	Cinnamyl formate*	104-65-4	-	-	-	-	-	0.19±0.03
59	Hexyl benzoate*	6789-88-4	0.30±0.06	0.21±0.03	0.16±0.14	0.13±0.07	0.51±0.06	0.11±0.11
60	3-Buten-2-one, 4-phenyl-*	1896-62-4	0.08±0.02	0.08±0.05	0.05±0.04	0.14±0.05	0.05±0.03	0.04±0.03
61	Alkane2		1.07±0.14	0.26±0.12	0.23±0.11	0.38±0.10	0.32±0.16	0.05±0.06
62	3-Hexen-1-ol, benzoate, (Z)-	25152-85-6	0.52±0.06	0.38±0.10	0.12±0.04	0.45±0.13	0.82±0.11	0.15±0.06
63	(E)-Cinnamyl acetate	21040-45-9	1.28±0.47	1.48±0.83	50.96±8.52	0.34±0.15	0.41±0.08	52.22±3.68
64	Eugenol	97-53-0	64.87±3.39	43.03±3.47	48.80±3.25	45.79±5.18	56.94±5.06	34.62±4.05
65	unknown 12		0.50±0.15	-	-	-	-	-
66	Nonanoic acid	112-05-0	0.08±0.02	0.22±0.19	0.14±0.03	0.06±0.02	0.15±0.10	0.14±0.08
67	(E)-Methyl isoeugenol	6379-72-2	0.73±0.16	1.51±0.27	0.61±0.37	0.56±0.17	0.75±0.11	0.49±0.10
68	unknown 13		0.33±0.16	0.02±0.03	-	0.05±0.04	0.03±0.05	0.01±0.03
69	unknown 14		0.22±0.03	0.51±0.06	0.37±0.06	0.53±0.15	0.51±0.06	0.27±0.07
70	Alkane3		14.10±2.72	4.92±0.86	6.30±1.00	9.13±2.75	8.86±2.65	3.97±0.99
71	cis-Isoeugenol	5912-86-7	0.13±0.02	0.20±0.10	0.03±0.02	0.09±0.05	0.09±0.06	0.04±0.03
72	unknown 15		-	0.26±0.06	-	0.05±0.05	-	-
73	(E)-Cinnamic alcohol	4407-36-7	0.44±0.08	0.18±0.09	36.33±6.25	0.11±0.08	0.30±0.07	40.00±7.31
74	unknown 16		0.42±0.09	0.34±0.13	0.17±0.06	0.34±0.12	0.44±0.13	0.17±0.08

75	unknown 17		0.01±0.03	0.04±0.04	-	0.03±0.02	0.04±0.03	0.00±0.01
76	4-allylphenol	501-92-8	40.04±9.10	15.53±1.98	7.52±2.89	9.88±3.79	34.19±5.28	10.92±2.07
77	trans-Isoeugenol	5932-68-3	0.42±0.04	0.26±0.02	0.27±0.02	0.30±0.07	0.28±0.07	0.22±0.02
78	unknown 18		0.01±0.03	-	-	0.01±0.01	0.00±0.01	-
79	Benzyl ether*	103-50-4	0.11±0.03	0.28±0.10	0.23±0.05	0.04±0.02	0.17±0.10	0.23±0.11
80	5-Indanol*	1470-94-6	0.04±0.01	-	0.03±0.03	0.14±0.05	0.04±0.03	0.06±0.04
81	Alkane4		2.07±0.14	0.99±0.21	0.96±0.38	1.69±0.59	1.07±0.65	1.42±0.48
82	unknown 19		7.84±1.82	14.20±1.23	5.61±1.13	11.89±1.97	10.22±3.71	6.31±0.92
83	2-Allylphenol*	1745-81-9	0.30±0.06	0.06±0.01	0.02±0.02	0.07±0.14	0.13±0.01	0.13±0.08
84	unknown 20		0.39±0.23	0.25±0.20	-	0.01±0.03	0.30±0.30	0.06±0.12
85	unknown 21		0.16±0.07	0.08±0.03	0.08±0.10	0.32±0.31	0.24±0.09	0.28±0.13
86	unknown 22		1.06±0.19	1.65±0.39	0.67±0.21	0.90±0.24	1.69±0.90	0.70±0.32
87	unknown 23		0.15±0.03	0.07±0.06	0.05±0.07	0.09±0.06	0.24±0.07	0.14±0.08
88	Benzyl Benzoate	120-51-4	63.29±11.21	40.94±4.13	37.39±5.58	54.74±9.51	64.67±16.78	35.52±9.53
89	unknown 24		0.27±0.09	0.10±0.13	0.07±0.12	0.41±0.64	0.08±0.07	0.10±0.17
	Total		607.94	481.89	541.37	520.47	480.34	562.90
	Total of the identified		595.70	463.28	533.52	505.07	465.54	554.21
	Identified/Total		97.99%	96.14%	98.55 %	97.04%	96.92%	98.46%

"*" indicates that the substance may also be other isomers of the compound. "-" means that the substance is not detected in all samples of this cultivar.

Table S2. OAV s of 65 identified volatile compounds in six cultivars of *P. mume* blossoms

No.	Compound Name	CAS-number	Aroma Discription[a]	Olfactory Threshold/ (μg/g) [e]	OAV					
					YD	JM	GLH	LE	DFZS	GF
1	Dimethyl sulfide	75-18-3	cabbage, sulfur, gasoline	0.005	8.93±2.22	0.00±0.00[n]	7.57±6.76	11.25±16.76	3.40±1.69	5.04±1.90
2	Acetic acid, methyl ester	79-20-9	fruit, fresh, rum[b]	1.5	0.10±0.02	0.17±0.03	0.18±0.03	0.06±0.01	0.14±0.02	0.10±0.01
3	Hexanal	66-25-1	grass, fat	0.0036	58.67±5.93	45.04±14.66	22.58±7.55	29.85±5.10	14.74±6.75	38.98±7.99
4	o-Xylene	108-38-3	plastic	1	0.04±0.04	0.00±0.00	0.00±0.00	0.00±0.00	0.00±0.00	0.00±0.00
5	2-Hexenal, (E)-	6728-26-3	apple, green	0.04	14.67±1.75	20.47±5.57	8.40±1.57	5.65±0.80	10.94±5.20	12.92±4.57
6	Styrene	100-42-5	spice, gasoline	0.12	0.13±0.38	0.00±0.00	0.76±0.26	0.03±0.09	0.13±0.11	0.55±0.33
7	1-Hexanol	111-27-3	resin, flower, green	0.7	0.13±0.03	0.07±0.02	0.05±0.01	0.07±0.04	0.07±0.03	0.09±0.02
8	3-Hexen-1-ol*	544-12-7	moss, fresh	1.63	0.09±0.02	0.03±0.01	0.03±0.03	0.05±0.02	0.04±0.02	0.05±0.02
9	2-Hexen-1-ol（E）	928-95-0	green, leaf, walnut	0.1	1.49±1.33	2.08±0.44	1.14±0.37	0.68±0.25	1.07±0.46	1.38±0.39
10	Nonanal	124-19-6	fat, citrus, green	0.00425	61.63±8.28	55.18±6.48	85.73±10.31	44.52±8.05	59.12±10.25	126.52±21.02
11	Acetic acid	64-19-7	acid	1.2	0.06±0.05	0.07±0.02	0.08±0.02	0.05±0.01	0.03±0.01	0.08±0.04
12	6-Hepten-1-ol, 2-methyl-*	67133-86-2	-	-	-	-	-	-	-	-
13	Benzaldehyde	100-52-7	sharp, sweet, bitter, almond, cherry	0.2	785.00±46.04	560.00±21.04	540.10±30.02	633.82±49.13	313.06±37.98	749.56±40.27
14	2-Nonenal*	2463-53-8	fatty, green, waxy, cucumber, melon	0.0005	132.79±112.21	0.00±0.00	198.28±97.27	0.00±0.00	0.00±0.00	65.09±78.30
15	Fenchol*	1632-73-1	camphoreous, pine, woody, dry, rooty	-	-	-	-	-	-	-
16	Bornyl acetate*	76-49-3	woody, pine, herbal,	0.075[f]	0.90±0.79	0.00±0.00	0.03±0.09	0.00±0.00	0.00±0.00	0.09±0.26

17	Methyl benzoate	93-58-3	cedar, spicy phenolic, wintergreen, almond, floral, cananga, odorata	0.00052	331469.15±13902.30	398572.02±13170.47	320964.28±11343.53	405298.09±22440.74	408084.40±12208.62	303542.79±16951.69
18	(E)-α-Elemene*	5951-67-7	-	-	-	-	-	-	-	-
19	1-Nonanol	143-08-8	fat, green	0.002	41.03±8.16	88.09±14.79	0.00±0.00	34.21±7.31	84.66±20.37	56.28±20.90
20	Ethyl benzoate	93-89-0	fruit, musty, sweet, wintergreen	0.053	5.18±1.99	21.30±7.17	44.94±9.24	10.14±5.07	8.78±2.66	22.88±14.93
21	4-Allylanisole	140-67-0	sweet, phenolic, anise, spicy	0.006	83.56±19.62	21.51±8.64	37.03±14.03	18.63±16.53	199.70±24.50	31.06±12.13
22	Benzyl formate	104-57-4	floral, fruity, spicy, almond, cranberry	0.177[g]	0.00±0.00	0.28±0.22	0.45±0.42	0.52±0.38	0.00±0.00	0.38±0.51
23	Borneol*	507-70-0	pine, woody, camphoreous, balsamic	0.14[h]	0.76±0.14	1.25±0.74	0.61±0.16	0.51±0.24	0.85±0.25	0.96±0.23
24	Benzyl acetate	140-11-4	sweet、floral、fruit、Jasmine、fresh	0.002	7993.82±604.97	4345.63±1073.17	2168.16±191.01	5643.68±812.39	418.15±74.81	2901.00±579.78
25	Carvone*	99-49-0	minty, licorice	0.027	3.28±0.66	0.00±0.00	0.00±0.00	0.00±0.00	0.00±0.00	0.00±0.00
26	(Z,E)-α-Farnesene*	26560-14-5	-	-	-	-	-	-	-	-
27	Methyl salicylate	119-36-8	peppermint	0.06	2.01±0.21	8.35±1.03	4.95±1.27	6.00±1.91	4.15±0.48	6.86±1.20
28	α-Farnesene*	502-61-4	woody、sweet	0.016[i]	167.07±51.21	0.00±0.00	0.00±0.00	0.00±0.00	0.00±0.00	0.00±0.00
29	3,4-Dimethoxytoluene*	494-99-5	stale、musty	0.005	55.44±5.31	15.88±7.43	26.54±9.28	102.29±13.83	25.89±9.85	37.98±24.99
30	2,4-Decadienal*	2363-88-4	orange, sweet,	0.0003	158.53±134.1	0.00±0.00	0.00±0.00	0.00±0.00	0.00±0.00	0.00±0.00

			fresh, citrus, fatty		0				
31	Carveol*	1197-07-5	caraway、solvent	0.25	0.30±0.35	0.00±0.00	0.00±0.00	0.00±0.00	0.00±0.00	0.00±0.00
32	Benzyl alcohol	100-51-6	floral、rose、phenolic、spice	5.5	7.53±1.04	2.51±0.48	3.58±0.58	4.30±0.64	1.24±0.24	4.12±0.50
33	Butyl benzoate	136-60-7	spice	-	-	-	-	-	-	-
34	(Z)-Cinnamaldehyde	57194-69-1	spicy, cinnamon	-	-	-	-	-	-	-
35	Phenylethyl Alcohol	1960/12/8	honey、spice、rose,、lilac	41.1	0.00±0.00	0.00±0.00	0.00±0.00	0.00±0.00	0.00±0.00	0.00±0.00
36	Isoamyl benzoate	94-46-2	honey、sweet[c]	0.25	0.94±0.11	3.87±0.76	1.23±0.44	2.24±0.43	6.41±1.00	1.68±0.52
37	Creosol	93-51-6	spicy, clove, vanilla, phenolic	0.021	13.97±0.98	0.00±0.00	6.02±8.68	0.00±0.00	0.00±0.00	0.00±0.00
38	Phenylpropyl acetate	122-72-5	sweet, balsamic, storax, spicy, cinnamon	-	-	-	-	-	-	-
39	trans-β-Ionone	79-77-6	seaweed、violet、flower、raspberry	0.000007	64831.31±16666.15	58367.53±4829.57	33826.24±22943.26	30862.43±8876.74	21560.30±11394.21	37296.25±11507.64
40	Dihydro-β-ionol	3293-47-8	woody、floral, amber	0.031[j]	7.19±1.63	2.75±0.55	0.99±1.85	2.17±1.01	0.41±0.97	2.16±1.51
41	Pentyl benzoate	2049-96-9	floral, green, musty, amber, balsamic	-	-	-	-	-	-	-
42	Methyleugenol	93-15-2	sweet、fresh、warm、spicy、clove、carnation、cinnamon	0.068	75.12±8.35	22.54±5.58	71.10±4.42	29.00±9.80	38.22±8.49	30.26±4.79
43	Alkane1									

44	3-Phenyl-1-propanol	122-97-4	sweet, spicy, cinnamyl, floral, hyacinth	0.551[k]	0.33±0.20	0.00±0.00	2.01±1.21	0.00±0.00	0.13±0.39	2.19±0.38
45	(E)-Cinnamaldehyde	14371-10-9	sweet、spicy、candy、cinnamon、warm	6	0.15±0.05	0.04±0.02	4.59±1.23	0.05±0.03	0.32±0.05	4.18±0.41
46	(E)-Methyl cinnamate	1754-62-7	strawberry	0.12[l]	1.97±0.29	3.43±0.69	3.35±0.66	2.47±0.38	7.12±0.82	2.35±0.53
47	Cinnamyl formate*	104-65-4	balsamic, fruity, floral, green, herbal, cinnamyl	-	-	-	-	-	-	-
48	Hexyl benzoate*	6789-88-4	fresh, balsamic, sappy, clean, woody	-	-	-	-	-	-	-
49	3-Buten-2-one, 4-phenyl-*	1896-62-4	-	-	-	-	-	-	-	-
50	Alkane2									
51	3-Hexen-1-ol, benzoate, (Z)-	25152-85-6	fresh, green, leafy, floral, orchid, balsamic, fatty	-	-	-	-	-	-	-
52	(E)-Cinnamyl acetate	21040-45-9	sweet、floral、spicy、spice	0.15	8.56±3.17	9.88±5.57	339.73±56.80	2.24±0.98	2.76±0.52	348.16±24.56
53	Eugenol	97-53-0	sweet、spicy、clove、woody	0.007	9267.71±483.68	6147.39±496.08	6971.31±464.79	6540.78±739.34	8133.62±722.27	4946.27±578.28
54	Nonanoic acid	112-05-0	green、fat	3	0.03±0.01	0.07±0.06	0.05±0.01	0.02±0.01	0.05±0.04	0.04±0.03
55	(E)-Methyl isoeugenol	6379-72-2	spicy[d]	-	-	-	-	-	-	-

56	Alkane3									
57	cis-Isoeugenol	5912-86-7	sweet、woody、roast	6[m]	0.02±0.00	0.03±0.02	0.01±0.00	0.01±0.01	0.02±0.01	0.01±0.01
58	(E)-Cinnamic alcohol	4407-36-7	sweet、spice、hyacinth、spicy、cinnamon	0.077	5.70±1.06	2.34±1.16	471.77±81.23	1.39±1.00	3.89±0.89	519.48±94.88
59	4-allylphenol	501-92-8	phenolic、medicinal、herbal	0.019[k]	2107.63±479.13	817.17±104.23	395.70±151.85	519.90±199.47	1799.25±278.14	574.55±109.20
60	trans-Isoeugenol	5932-68-3	floral	-	-	-	-	-	-	-
61	Benzyl ether*	103-50-4	sweet, fruity, cherry, earthy, mushroom	-	-	-	-	-	-	-
62	5-Indanol*	1470-94-6	-	-	-	-	-	-	-	-
63	Alkane4									
64	2-Allylphenol*	1745-81-9	-	-	-	-	-	-	-	-
65	Benzyl Benzoate	120-51-4	sweet、spice、floral、fruit	0.341	185.60±32.88	120.07±12.12	109.64±16.37	160.53±27.89	189.64±49.21	104.17±27.94

[a]Aroma Descriptions were from the database (www.perflavory.com)(accessed on 6 April 2022) unless otherwise noted[26]. [b]From Ln Chen(2021)[29]. [c]From Jb Liu(2013)[30]. [d]From Jy Chen(2018)[31]. [e]Olfactory thresholds were from *Compilations of Flavour Threshold Values in Water and Other Media* unless otherwise noted[23]. [f]From Nian Zhang(2022)[32]. [g]From Xr Li(2021)[33]. [h]From Hl Miao(2009)[34] . [i]From Hq Liu(2022)[35]. [j]From Yl Jin(2021)[36]. [k]From LH Ma(2021)[24]. [l]From Zl Wang(2021)[37]. [m]From Jd Wu(2020)[38]. [n]0 means OAV≈0 taking two decimal.
